# Optimal timing for initiation of antiretroviral therapy: a prospective study on treatment naive HIV patients

**DOI:** 10.1186/1471-2334-12-S1-P38

**Published:** 2012-05-04

**Authors:** Jaya Chakravarty, Avinash Singh, Anup Singh, Madhukar Rai, Anoop Gupta, Amit Agarwal, Shyam Sundar

**Affiliations:** 1 Department of Medicine, Institute of Medical Sciences, Banaras Hindu University, Varanasi, UP, India

## Background

Early institution of therapy has been advocated worldwide. However, until recently, NACO guidelines recommended therapy in HIV positive patients with CD4 count <250/µL. In this study we observed the clinical and immunological disease progression for a year in treatment naïve patients with CD4 count between 250-500/µL.

## Method

150 treatment naïve adults PLHIV with CD4 count between 250 -500/mm^3^ in WHO clinical stage 1 or 2 were included in the study and enrolled into 2 groups at 2:1 ratio and followed for 1 year. Group A with CD4 count between 250-350/mm^3^ & Group B with CD4 count between 350-500/mm^3^. At the end of study each group was evaluated for number of opportunistic infection, ART conversion, conversion to stage III and IV, lost to follow up (LFU) and death. Statistical analysis was done by SPSS 16.0 version. Data were expressed as mean (±SD) for continuous variable and percentage for categorical variable. An independent sample t-test was used to detect differences in clinical and laboratory results.

## Result

As compared to Group B, Group A had significantly higher number of opportunistic infection (27% vs 8%, p=0.006), ART conversion (30% vs 10%, p=0.012), death (9% vs 0%, p=0.021) and LFU (20% vs 6% p=0.037).

## Conclusion

This study reinforces the need of early initiation of treatment in patients with CD4 <350/ µL.

